# Reduced expression of peroxisome proliferator-activated receptor alpha in BAL and blood T cells of non-löfgren’s sarcoidosis patients

**DOI:** 10.1186/s12950-015-0071-6

**Published:** 2015-04-09

**Authors:** Muntasir Abo Al Hayja, Anders Eklund, Johan Grunewald, Jan Wahlström

**Affiliations:** Department of Medicine and CMM, Respiratory Medicine Unit, Karolinska Institutet and Karolinska University Hospital Solna, Stockholm, Sweden; Lung Research Laboratory L4:01, Karolinska University Hospital Solna, S-171 76 Stockholm, Sweden

**Keywords:** Peroxisome proliferator-activated receptors α, β/δ and γ, Sarcoidosis, Bronchoalveolar lavage (BAL), Flow cytometry, BAL and blood CD4^+^- and CD8^+^ T lymphocytes, Alveolar macrophages, RT-PCR

## Abstract

**Background:**

Sarcoidosis is a granulomatous disease affecting in particular the lungs. The peroxisome proliferator-activated receptors (PPARs) play important regulatory roles in inflammation. The aim of this study was to gain more insight about the expression of all three PPARs (α, β/δ and γ) in sarcoidosis.

**Methods:**

Bronchoalveolar lavage (BAL) cells and peripheral blood cells were obtained from healthy controls (HC) and sarcoidosis patients with Löfgren’s syndrome (LS) and without Löfgren’s syndrome (non-LS). PPARs mRNA expression was analyzed in total BAL cells and in FACS (Fluorescence-activated cell sorting) sorted alveolar macrophages (AM) and CD4^+^ T cells respectively by comparative RT-PCR. PPARs protein expression was analyzed in AM, and in BAL and blood CD4^+^ and CD8^+^ T cells by flow cytometry.

**Results:**

In BAL CD4^+^ T cells, we noticed a significantly lower PPARα mRNA expression in sarcoidosis patients compared with HC. In non-LS patients, a significantly lower PPARα protein expression in BAL CD4^+^ T cells was detected as compared with LS patients. In peripheral blood CD4^+^ T cells, non-LS patients had a significantly lower expression of PPARα and PPARγ compared with LS patients.

**Conclusion:**

The lower protein expression of PPARα and PPARγ could contribute to the persistent T-cell driven inflammation noted especially in non-resolving sarcoidosis, common in non-LS patients.

**Electronic supplementary material:**

The online version of this article (doi:10.1186/s12950-015-0071-6) contains supplementary material, which is available to authorized users.

## Introduction

Sarcoidosis is a systemic disease characterized by noncaseating granulomas affecting many organs, in particular the lungs and the intrathoracic lymph nodes [1]. Sarcoidosis is a multifactorial [2] disease, where environmental factors and several genetic loci contribute to the pathogenesis of the disease [3]. Moreover, some studies have suggested that sarcoidosis is associated with increased risk of cancer [4,5]. The cause of sarcoidosis remains elusive. Evidence points towards that sarcoidosis is an exaggerated immune response to persistent antigens from not completely degraded bacteria such as mycobacteria and propionibacterium acnes [1].

In sarcoidosis, the interaction between antigen presenting cells (APCs) and an unknown antigen(s) leads to a polarized T-helper 1 (Th1) CD4^+^ response including production of Th1 cytokines such as interferon-gamma (IFN-γ), tumor necrosis factor-α (TNF-α) and interleukin-2 (IL-2) [1], and the formation of granulomas. Pulmonary sarcoidosis is characterized by an increased number of CD4^+^ T cells in bronchoalveolar lavage fluid (BALF). Analyses of the T Cell Receptor (TCR) suggest oligoclonal expansion of the CD4^+^ T cells using particular Vα or Vβ genes [3]. In most cases the granulomas resolve, but in some cases the local environment of the granulomas may change to promote the development of fibrosis [1].

The clinical presentation of sarcoidosis ranges from an incidental chest radiographic finding in an asymptomatic patient to chronic progressive organ dysfunction. A distinct subgroup of sarcoidosis patients is Löfgren’s syndrome (LS), characterized by an acute onset with bilateral hilar lymphadenopathy and in some cases parenchymal infiltrates, erythema nodosum and/or bilateral ankle arthritis or periarticular inflammation, and usually fever [3]. Patients with LS often have spontaneously resolving disease, while patients without LS (non-LS) often present with a more gradual, insidious onset, and are more likely to develop chronic disease. Studies from our laboratory of Swedish patients with sarcoidosis demonstrated a strong correlation between the carriage of HLA-DRB1*0301 (DR3^+^) and the development of LS and with disease resolution [3].

The peroxisome proliferator-activated receptors (PPARs) play important regulatory roles in numerous cellular processes related to metabolism, differentiation, proliferation, and inflammation. PPARs are a subgroup of the nuclear receptor (NR) superfamily. Three PPARs have been described, which are designated as α, β/δ and γ forms. The PPARs are expressed in many cell types, including macrophages and T lymphocytes [6,7]. All three PPARs subtypes can be activated by a variety of polyunsaturated long-chain fatty acids, prostaglandins, leukotrienes, and by many synthetic compounds [8]. PPAR*α* is specifically activated by fibrates (used to treat hypertriglyceridemia and hyperlipidemia), whereas PPARγ is activated by glitazones (used to treat diabetes type II). PPARs regulate gene expression after binding as a heterodimer with the retinoid X receptors (RXRs) and accumulation in the nucleus. A major role for PPARs is the trans-suppression of inflammatory gene activation by interfering with the NF-κB, STAT-1, and AP-1 transcription complexes without direct binding to the DNA [9]. PPARα PPARβ/δ and PPARγ ligands have been reported to inhibit Th1 pro-inflammatory cytokine production by activated T cells [10,11].

PPARα and PPARγ are recognized as potentially important players in immunomodulation and anti-inflammatory regulation in different inflammatory disorders including pulmonary diseases such as COPD and asthma [6]. To date, the expression and function of PPARs in pulmonary sarcoidosis is scantily documented. Alveolar macrophages (AM) from patients with active chronic sarcoidosis exhibited a deficiency of PPARγ activity, and PPARγ gene expression in BAL cells was reduced in patients with severe disease [12,13]. To our knowledge, the expression of PPARα and PPARβ/δ in sarcoidosis has not been documented.

Considering the regulatory and immunomodulatory actions of PPARs, we hypothesized that down regulation of their expression in sarcoidosis may represent a mechanism leading to the promotion of inflammation. The aim of this study was to gain more insight about the expression of all three PPARs (α, β/δ and γ) in human AM and CD4^+^ T cells in LS and non-LS patients in comparison to healthy controls (HC).

## Methods

### Study subjects

All included patients in this study were referred to the Lung and Allergy clinic (at Karolinska University Hospital or at Stockholm South General Hospital, Stockholm, Sweden) for an investigational work up of clinically suspected pulmonary sarcoidosis. The diagnosis of sarcoidosis in the non-Löfgren patients was established by clinicoradiographic findings compatible with the disease and in most cases by biopsies showing non-caseating epithelioid granuloma. If biopsies were negative despite typical clinicoradiographic findings the diagnosis was considered likely if BALF analysis showed a CD4^+^/CD8^+^ ratio > 4 and the diagnosis remained the same at follow-ups. In addition, other causes of sarcoid like diseases and reactions were reasonably excluded [14,15]. In Löfgren patients the diagnosis of presumptive sarcoidosis was made with high confidence based on typical clinical findings, provided there are no other explanations for the symptoms. Furthermore, the patients had BALF with a high percentage of lymphocytes and a CD4^+^/CD8^+^ ratio > 4 [16]. Written informed consent was obtained from all subjects, and the Regional Ethical Review Board (Stockholm, Sweden) approved the study.

A total of 29 healthy controls (HC) and 57 sarcoidosis patients were sequentially recruited to this study (clinical and BAL cells characteristics are summarized in Table 1). All HC were non-smokers, had normal lung function results, unremarkable chest X-ray, and none of them showed any signs of lower respiratory tract infection at least six weeks prior to the bronchoscopy. All patients had a clinical picture completely consistent with sarcoidosis, and they were subdivided into two groups according to their clinical phenotypes: Löfgren’s syndrome (LS) and non-LS. Patients were also classified as being HLA allele DRB1*0301 (DR3) positive or DR3 negative. Twenty-seven patients had LS, (74% DR3^+^, median age 39 years (min-max: 28–56, 15 females and 12 males)). Thirty patients had non-LS (80% DR3^−^, median age 47 (min-max: 27–71, 9 females and 21 males)). Only patients, who did not receive anti-inflammatory or immunosuppressive medications, were included into the study. None of the patients included in this study was on treatment with fibrates or glitazones.

**Table 1:** **Characterization of sarcoidosis patients and HC**

	**Löfgren’s**	**Non-Löfgren’s**	**Healthy controls**
**Subjects number**	27	30	29
**Age**	39 (28–56)****	47 (27–71)****	26 (18–42) ¤
**Sex, female/male**	15/12	9/21	15/14
**smoking history (NS/FS/OS/CS)**	20/5/2/0	16/13/1/0	29/0/0/0
**Radiograph stages (0,I, II, III, IV, ND)**	0/16/9/0/0/2	0/7/9/4/4/6	29/0/0/0/0
**HLA (DR3+/DR3-/ND)**	20/6/1	3/24/3	ND
**BAL analysis**			
Recovery %	68 (44–80)	63,5 (44–84)	72 (34–88)
Viability %	95 (85–99)	95 (78–98)	95 (88–98)
Cell concentration (*10^6^/L)	225 (49–588)****	188 (83,5-476)****	90,5 (29–167) ¤
Total cell number (*10^6^)	36 (9–77)****	28 (10–81)***	16 (5–31,5) ¤
**BAL differential cell counts**			
% Macrophages	68 (39,4-91)****	71 (39,70-85)****	87 (60,8-97,2) ¤
% Lymphocytes	30 (8,5-57,8)****	26 (13–58,2)****	11 (2,4-33,7) ¤
% Neutrophils	1,0 (0–5,8)	1,0 (0–6)	1,8 (0,2-6)
% Eosinophils	0,3 (0–2,5)	0,3 (0–4,4)	0,2 (0–1,6)
CD4/CD8 ratio	9,6 (1,1-28)****	5,8 (1,6 -33)***	2,65 (0,9-4,8) ¤
**Pulmonary function tests**			
FVC (% of predicted value)	90 (67–120)****	84 (50–105)****	111 (86–130) ¤
FEV1 (% of predicted value)	87 (61–104)***	82 (46–112)****	107 (86–125) ¤
TLC (% of predicted value)	92 (82–123)**	85 (62–186) #	ND
DLCO (% of predicted value)	86 (66–126)	82 (54–130)	ND

Data are presented as median (minimum-maximum). NS: never smoker, FS: former smoker, OS: occasional smoker, CS: current smoker. Radiographic stages 0: normal, stage I: bilateral hilar lymphadenopathy (BHL), stage II: BHL and parenchymal infiltrates, stage III: parenchymal infiltrates without BHL, stage IV: signs of fibrosis. BAL: bronchoalveolar lavage; VC: vital capacity; FEV_1_: forced expiratory volume in 1 s; *D*LCO: diffusing capacity of the lung for carbon monoxide; ND: not determined; ¤ Kruskal-Wallis test was used to compare all three groups and a significant difference was only observed when LS and non-LS patients were compared with HC; # Mann–Whitney *U*-test between LS and non-LS patients; **: p < 0.01, ***: p < 0.001, ****: p < 0.0001.

The study progressed through three sequential stages. In the first stage, the expression of PPARs mRNA in total BAL cells was analyzed in 6 HC and 16 sarcoidosis (7 LS and 9 non-LS) patients. In the second phase, the expression of PPARs mRNA in FACS (Fluorescence-activated cell sorting) sorted BAL CD4^+^ T lymphocytes and AM were analyzed separately in a total 19 HC and 34 sarcoidosis (17 LS and 17 non-LS) patients. In the last stage, PPARs protein expression in BAL and blood CD4^+^- and CD8^+^ T lymphocytes and AM was investigated by using flow cytometry. Six HC and nine sarcoidosis patients (4 LS and 5 NLS) were recruited for this flow cytometry analysis. Due to limitations in numbers of BAL and blood cells available for this study, some samples of patients and HC could not be included in all types of analyses. However, to ensure no selection bias, all individuals were randomly included in the different experiments. The exact numbers of controls and patients in each group are indicated in the figures.

### Bronchoalveolar lavage (BAL) and preparation of cells

BAL was performed as previously described [17]. Briefly, after light sedation and topical local anesthesia, a flexible fiber-optic bronchoscope (Olympus Optical Co., Tokyo, Japan) was passed trans-nasally and BAL was performed in a subsegmental bronchus in the middle-lobe. BAL was then performed by sequentially instilling and gently suctioning 50 ml of warmed (37°C) and sterile phosphate-buffered saline (PBS) solution five times. The BAL fluid aliquots were pooled and collected in a siliconized plastic vessel kept on ice at 4°C. The recovered volume as percentage of total instilled fluid is indicated in Table 1. The BALF was gently passed through a Dacron gauze (Milipore, Cork, Ireland) and centrifuged at 400 g for ten minutes at 4°C. The pellet was resuspended in PBS. The cells were counted in a Bürker chamber. The expression of cell surface antigens (CD3, CD4, CD8) and the CD4^+^ to CD8^+^ cell ratio were performed by flow cytometric analysis (BD FACSCanto II flow cytometer) using monoclonal antibodies against CD3^+^ (Pacific Blue Mouse Anti-human CD3, clone UCHT1; BD Biosciences), CD4^+^ (APC-H7 Anti-human CD4, clone SK3; BD Biosciences), CD8^+^ (AmCyan Anti-human CD8, cloneSK1; BD Biosciences) as previously described [18].

### Flow cytometric sorting of BAL macrophages and CD4^+^ T cells

BAL cells were stained with PE Mouse Anti-human CD4, clone RPA-T4. The stained cells were sorted by FACSVantage (BD Biosciences). BAL cells were gated on lymphocytes and macrophages and the stained CD4^+^ T cells and macrophages were effectively separated. The purity of the sorted population, which was determined by flow cytometry, was approximately 98%.

### Flow cytometric analysis of PPAR α, β/δ, γ

To detect intracellular PPAR α, β/δ, γ on a single cell level in blood and BALF cells, flow cytometry was utilized. The cells were fixed and permeabilized with the BD Pharmingen™ Transcription Factor Buffer set (BD Biosciences). The following primary antibodies and isotypes controls were added: Mouse monoclonal [3B6/PPAR] to PPAR alpha - ChIP Grade (ab2779), Mouse IgG2b [PLPV219] - Isotype Control (ab91366), Mouse monoclonal to PPAR delta (ab58137), Mouse IgG2a [ICIGG2A] - Isotype Control (ab91361), Mouse monoclonal [A3409A] to PPAR gamma 1 + 2 - ChIP Grade (ab41928) and Mouse IgG1 [ICIGG1] - Isotype Control (ab91353). The following secondary antibody was used: Goat polyclonal Secondary Antibody to Mouse IgG - H&L (DyLight® 488), pre-adsorbed (ab96879) (Abcam). Cell surface staining with anti- CD3, CD4, and CD8 was performed after blockade with mouse serum. Cells were analyzed on BD FACSCanto II flow cytometer (BD Bioscience) and data were processed using the web-based application Cytobank [19] (Cytobank, Inc). Alveolar macrophages and lymphocytes were gated based on characteristic light scatter properties. Within the lymphocyte gate, a further gate was set on CD3^+^ lymphocytes. Within the CD3^+^ gate, further gates were set on CD4^+^ and CD8^+^ cells to identify CD3^+^CD4^+^ and CD3^+^CD8^+^ T cells respectively. PPARs were then measured in CD4^+^ and CD8^+^ T cells and macrophages. Quantitative levels of PPARs, expressed as median fluorescence intensity (MFI), were determined after subtraction of the background level calculated from samples labelled with isotype control antibodies.

### RNA extraction and cDNA synthesis

Depending on the cells count (≤5×10^5^ or ≥ 10^6^) total RNA was isolated using the RNAqueous-micro kit or the RNAqueous kit (Ambion®) according to the manufacturer’s instructions. The quantity of total RNA was analysed using NanoDrop ND-1000Spectrophotometer (NanoDrop Technologies, Wilmington, DE, USA). To synthesize cDNA the high capacity RNA-to-cDNA kit (Invitrogen™) was used according to the manufacturer’s instructions.

### Analysis of PPAR α, β/δ, γ gene expression by real-time PCR

Real-time quantitative PCR (RT-PCR) was performed using gene-specific fluorogenic assays (TaqMan; Applied Biosystems) and an ABI Prism 7700 Sequence Detection System (Applied Biosystem). Proteasome Subunit, Beta Type, 2 (PSMB2) was used as a reference gene to normalize RT-PCR in BAL cells in sarcoidosis [20]. For relative quantification of PPARs gene expression in BAL cells, the comparative Ct method was used according to the formula 2^-∆∆Ct^ [21]. In the first step, the Ct of the target gene (PPAR α, β/δ, γ) was normalized to the endogenous control (PSMB2). In the second step, normalization to the median value of PPAR (α, β/δ, γ) gene expression in a HC group (calibrator sample) was performed. All samples were run in duplicates to ensure the validity of the experiment. The following primers were used: PSMB2 (Hs01002946_m1), PPARα (Hs00947539_m1), PPARδ (Hs00602622_m1), PPARγ (Hs00234592_m1) (Applied Biosystems).

### Statistical analysis

The nonparametric Mann–Whitney test or Kruskal-Wallis test was used to compare the distributions (medians) of two or three unmatched groups respectively. In the case of Kruskal-Wallis test, the Dunn’s post test was used to compare the difference between two groups. Wilcoxons signed ranks test was used to calculate differences in PPARs expression in BAL and blood CD4^+^ T cells. Values of p < 0,05 (*) were regarded as significant. All statistical tests were performed using the GraphPad PRISM version 6.04 (GraphPad Software, Inc).

## Results

### PPARs mRNA expression in BAL fluid and blood cells

**Figure 1:**
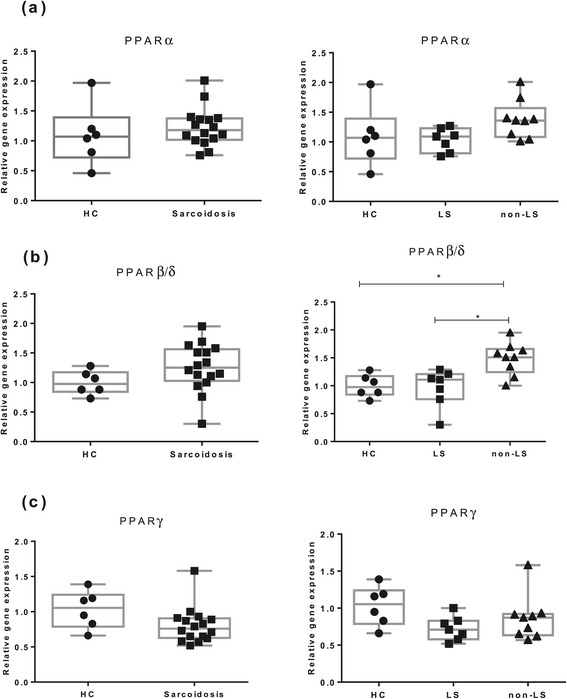
**The relative mRNA expression in total BAL cells of (a) PPARα, (b) PPARβ/δ and (c) PPARγ of HC (n = 6), patients with sarcoidosis (n = 16) and patients subgroups (LS = 7, non-LS = 9) are shown.** The boxes show median (25th-75th percentiles) values and the whiskers show minimum and maximum values. Each symbol represents an individual patient.

We first investigated PPARs mRNA expression levels in total BAL cells in HC and sarcoidosis patients (Figure 1). We found no significant differences in PPARα, PPARβ/δ and PPARγ mRNA expression between sarcoidosis patients and HC. An increased expression of PPARβ/δ mRNA was noted in non-LS patients compared with LS patients and HC (Figure 1b).

**Figure 2:**
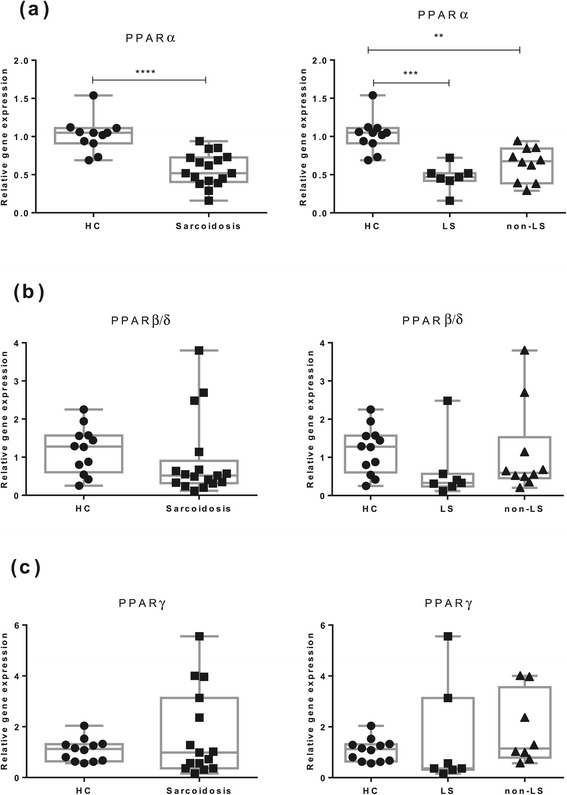
**The relative mRNA expression FACS-sorted BAL CD4** ^**+**^ **T cells of (a) PPARα, (b) PPARβ/δ and (c) PPARγ of HC (n = 12), patients with sarcoidosis (n = 17) and patients subgroups (LS = 7, non-LS = 10) are shown.** The boxes show median (25th-75th percentiles) values and the whiskers show minimum and maximum values. Each symbol represents an individual patient.

We then investigated mRNA expression in FACS sorted BAL CD4^+^ T cells (Figure 2). Here we noticed a significantly lower PPARα mRNA expression in sarcoidosis patients compared with HC (Figure 2a). This reduction was noted in both LS patients and non-LS patients.

**Figure 3:**
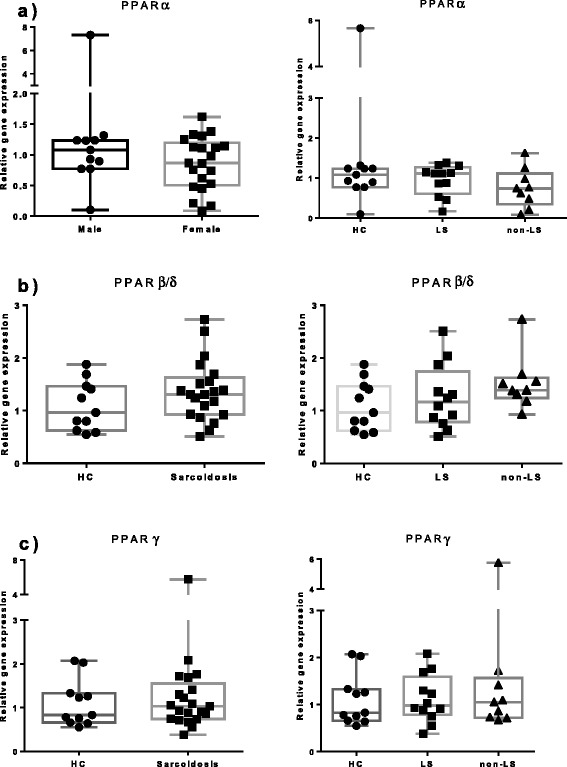
**The relative mRNA expression in FACS-sorted BAL macrophages of (a) PPARα, (b) PPARβ/δ** **and (c) PPARγ of HC (n = 11), patients with sarcoidosis (n = 21) and patients subgroups (LS = 12, non-LS = 9) are shown.** The boxes show median (25th-75th percentiles) values and the whiskers show minimum and maximum values. Each symbol represents an individual patient.

We also explored PPARs mRNA expression in FACS sorted AM (Figure 3). We noticed no significant differences in PPARα, PPARδ and PPARγ expression between sarcoidosis patients, including patient’s subgroups (LS and non-LS) and HC.

### Flow cytometric analysis of PPARs protein expression in blood and BAL cells

**Figure 4:**
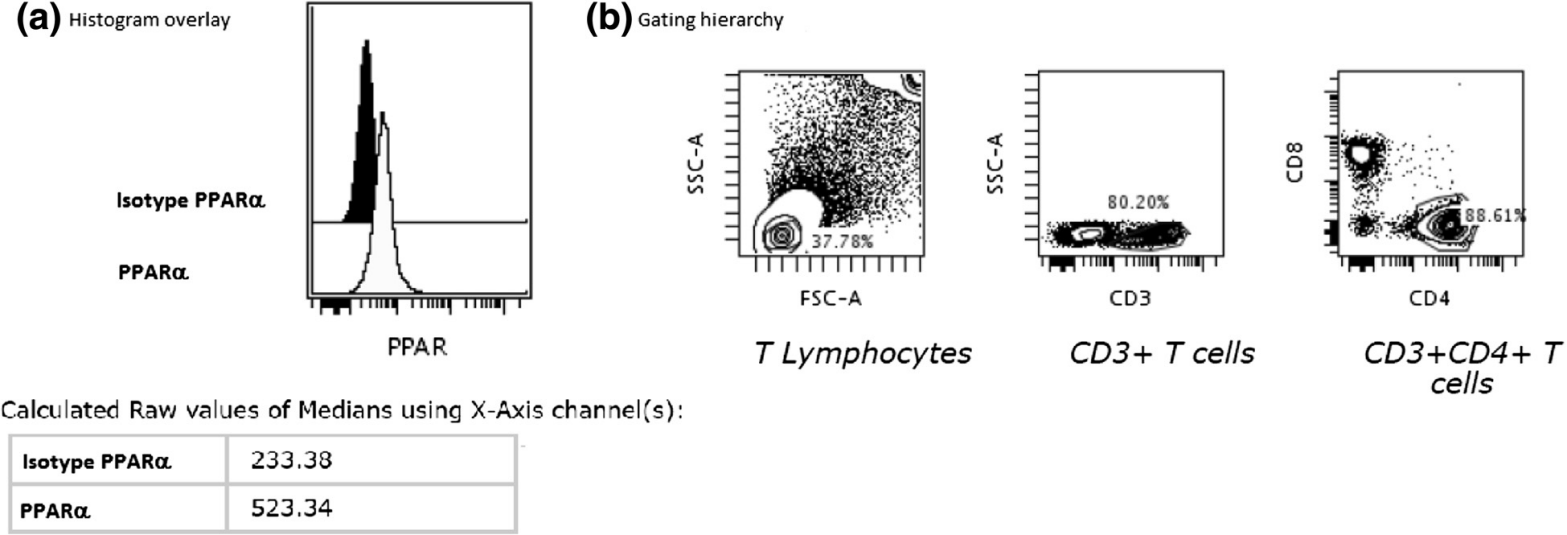
**Representative histogram overlay (a) -in a patient with LS syndrome- of PPARα expression in BAL CD4** ^**+**^ **T cells and the gating plots on CD4** ^**+**^ **T cells (b).** Black histogram is the isotype control; light gray histogram is the anti-PPARα. The x-axis of each histograms represents fluorescence intensity of the isotype and PPARα on a logarithmic scale, and the y-axis represents the total number of events. The values of the medians using the x-axis channels are presented in the table.

**Figure 5:**
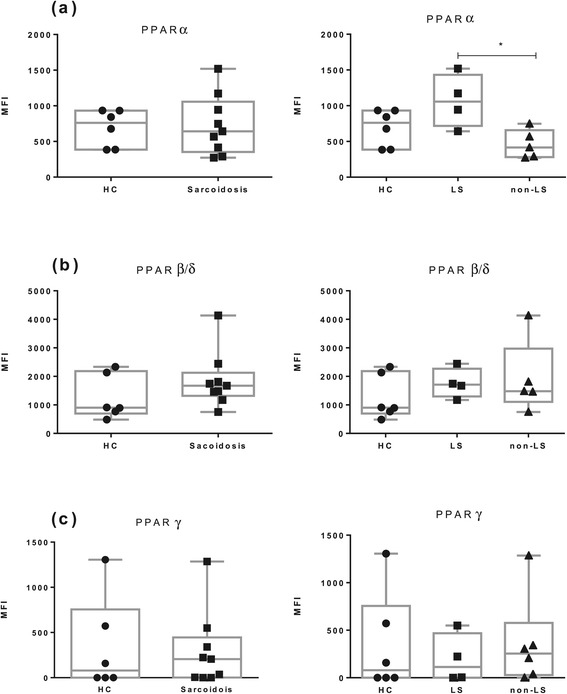
**Flow cytometric analysis of PPARs expression in BAL CD4** ^**+**^ **T cells. (a)** PPARα. **(b)** PPARβ/δ. **(c)** PPARγ. HC: (n = 6); LS patients (n = 4); non-LS patients (n = 5). MFI: median fluorescence intensity. The boxes show median (25th-75th percentiles) values and the whiskers show minimum and maximum values. Each symbol represents an individual patient.

An illustrative histogram overlay of PPARα expression in BAL CD4^+^ T cells in a patient with LS and the gating hierarchy is shown in (Figure 4). A significantly lower expression of PPARα in non-LS patients was detected as compared with LS patients (Figure 5a). This was observed also in the BAL CD8^+^ T subset (Additional file 1: Figure S1 a).

**Figure 6:**
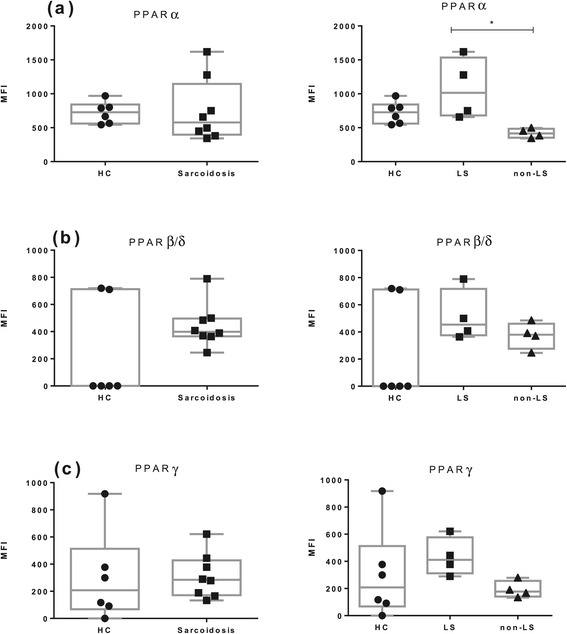
**Flow cytometric analysis of PPARs expression in peripheral blood CD4** ^**+**^ **T cells. (a)** PPARα. **(b)** PPARβ/δ. **(c)** PPARγ. HC (n = 6); LS patients (n = 4); non-LS patients (n = 4). MFI: median fluorescence intensity. The boxes show median (25th-75th percentiles) values and the whiskers show minimum and maximum values. Each symbol represents an individual patient.

In peripheral blood CD4^+^ T cells as well CD8^+^ T cells, non-LS patients had a significantly lower expression of PPARα as compared with LS patients (Figure 6a) and (Additional file 2: Figure S2 a). In both CD4^+^ and CD8^+^ T cells of peripheral blood, a strong trend toward a lower expression of PPARγ was also observed in non-LS patients as compared with LS patients (Figure 6c) and Additional file 2: Figure S2 c). This difference was statistically significant (p < 0.05) when directly comparing LS and non-LS patients.

**Figure 7:**
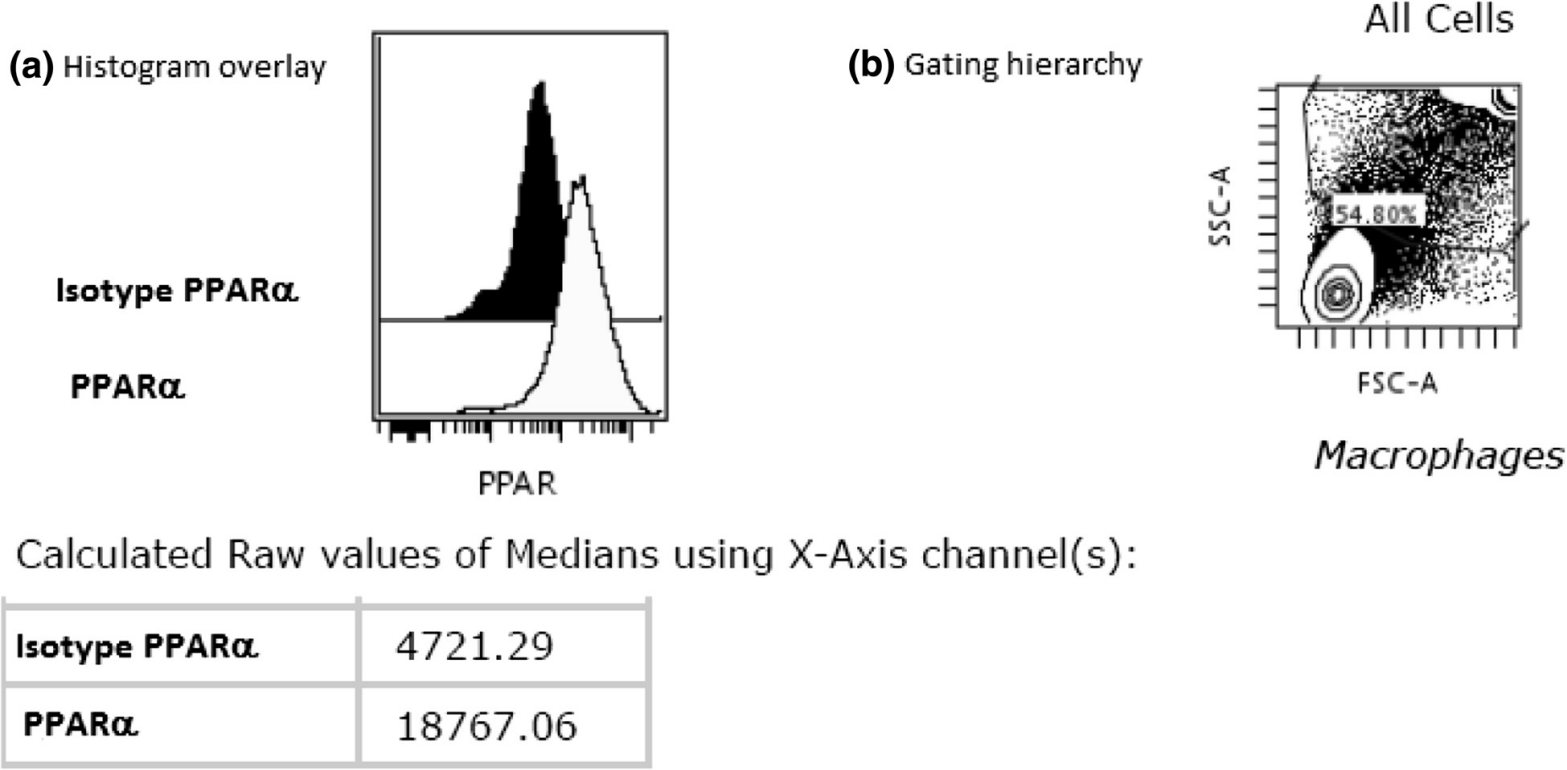
**Representative histogram overlay (a) of PPARα expression in alveolar macrophages and the gating of alveolar macrophages in a FSC-SCC dot (b).** Black histogram is the isotype control; light gray histogram is the anti-PPARα. The x-axis of each histograms represents fluorescence intensity of the isotype and PPARα on a logarithmic scale, and the y-axis represents the total number of events. The values of the medians using the x-axis channels are presented in the table.

**Figure 8:**
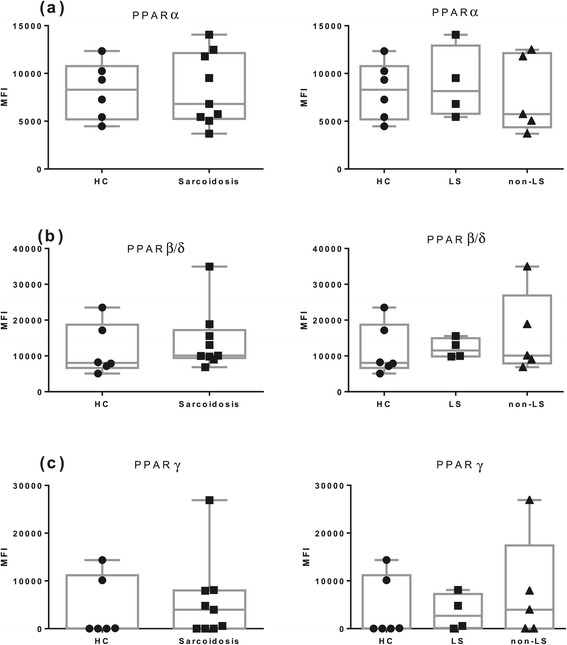
**Flow cytometric analysis of PPARs expression in alveolar macrophages. (a)** PPARα. **(b)** PPARβ/δ. **(c)** PPARγ. HC (n = 6); LS patients (n = 4); non-LS patients (n = 5). MFI: median fluorescence intensity. The boxes show median (25th-75th percentiles) values and the whiskers show minimum and maximum values. Each symbol represents an individual patient.

A representative histogram overlay of PPARα expression in AM and the related FACS plot of BAL cells with gating of AM is shown in (Figure 7). As shown in Figure 8, we did not observe any significant difference in PPARs expression in AM of sarcoidosis patients from HC.

### Comparison of PPARα, PPARβ/δ and PPARγ expression in BAL and blood CD4^+^ T cells in the same individual

Comparison of MFI between PPARα, PPARβ/δ and PPARγ expression in BAL and blood CD4^+^ T cells in the same individual revealed a statistically significantly lower expression of PPARβ/δ in blood CD4^+^ T cells as compared with BAL CD4^+^ T cells of healthy individuals. The same tendency was observed for PPARβ/δ expression in LS and non-LS patients (Additional file 3: Figure S3 b); the difference was statistically significant when all sarcoidosis patients were analyzed together. The expressions of PPARα, and PPARγ were comparable in BAL and blood CD4^+^ T cells in the same individual in all groups.

## Discussion

Recognizing that PPARs have an important role in controlling inflammatory responses [7], we investigated the expression of PPARs in total BAL cells, in BAL AM and in BAL and in peripheral blood CD4^+^ and CD8^+^ T cells from healthy controls (HC) and from sarcoidosis patients with or without Löfgren’s syndrome (LS and non-LS). Our data shows for the first time, that PPARα expression is downregulated in BAL and blood CD4^+^ and CD8^+^ T cells in non-LS patients. In addition, the expression of PPARγ was found to be reduced in blood CD4^+^ and CD8^+^ T cells in non-LS patients compared to LS patients.

These finding are consistent with prior published results of PPARα and PPARγ expression in other inflammatory disorders. PPARα expression was reported to be reduced in the inflamed lung of LPS- or allergen-exposed mice. Administration of fenofibrate (an PPARα agonist) increased PPARα expression while decreasing the inflammatory response [22]. Several studies showed that PPARα expression is reduced in inflamed skin [23]. Additionally, activation of PPARα with fenofibrate provides anti-inflammatory activity in allergic asthma [24]. Klotz et al., demonstrated that PPARγ protein expression is significantly reduced in peripheral blood mononuclear cells (PBMCs) in multiple sclerosis patients [25]. PPARγ expression is notably decreased in the colon of patients with ulcerative colitis [26]. Furthermore, PPARγ is down-regulated in lung tissue and epithelial cells of COPD patients [27].

Notably, PPARα and PPARγ ligand activation decreases the expression of inflammatory cytokines, such as IFNγ and TNFα by Th1 cells [10,11]. Previous studies from our group showed decreased expression of Th1 cytokines (INFγ and TNFα in BAL fluid cells in HLA-DR3^+^ sarcoidosis patients (typically with Löfgren’s syndrome) as compared with HLA-DR3^−^ patients [28]. Consequently, the reduced expression of PPARα in non-LS patients could contribute to the more pronounced Th1 response with elevated Th1 cytokines. The reduced expression of PPARα in CD4^+^ and CD8^+^ T cells in non-LS patients could contribute to a non-resolving disease and a worse prognosis in these patients compared to patients with LS.

Interestingly, PPARα-deficient T regulatory cells (Treg) lack suppressive capacity [29]. PPARα or PPARγ deficient mice showed reduced Treg recruitment to the sites of inflammation [30,29]. This could contribute to the reduced quantity of Treg cells in BALF observed in some studies [31,32] and the dysfunctional activity of these cells in sarcoidosis patients [33].

Th17 cells have been implicated also in sarcoidosis, although with conflicting results regarding their frequency [32,34,35], but the majority of BALF Th17 cells produce both IL-17 and INFγ (Th17 INFγ^+^) [32,36]. Th17 INFγ^+^ cells are thought to be pathogenic in several autoimmune diseases [37]. The activation of PPARα or γ reduces INFγ as well as IL-17 production in T cells [38,39]. In addition, PPARγ activation in CD4^+^ T cells selectively suppressed Th17 differentiation [40]. Thus, PPARα or PPARγ could be potential therapeutic targets in sarcoidosis.

Our study shows that the expression of PPARα mRNA is reduced in BAL CD4^+^ T cells in both LS and non-LS patients, but the expression of PPARα protein in LS patients is to some degree higher or at least comparable with the expression of PPARα protein in HC. This discrepancy highlights the importance of analyzing PPARs expression on both mRNA and protein level. RT-PCR assesses mRNA levels, which may be subject to post translational modifications that prevent subsequent protein production of functional proteins. In humans, the correlation between mRNA and protein concentration is very modest [41]. Our study utilizes flow cytometry to investigate the intracellular PPARs-protein expression at the level of an individual cell and the quantification of PPARs-protein by evaluation of MFI [42].

The role and expression of PPARβ/δ in inflammatory cells and in pulmonary disease is less well-established [6]. We found that PPARβ/δ mRNA expression is higher in total BAL cells in non-LS patients compared to LS patients and HC, and a tendency towards higher PPARδ protein expression in blood CD4^+^ and CD8^+^ T cells in sarcoidosis patients. N al Yacoub et al., showed that PPARδ is expressed both at mRNA and the protein level in human T cells, and that INF-α but not TCR stimulation causes increased expression of PPARδ mRNA in peripheral human T cells [11]. Again, ligand activated PPARδ repress the production of INFγ and IL-17 in T cells [43].

The similar levels of PPARs expression in both CD4^+^ and CD8^+^ T cells in this study are not unique for these transcription factors and have been documented for other transcription factors such as p-STAT1 and p-STAT6 [44].

Our data show that PPARs mRNA and protein expression in AM is comparable with HC in sarcoidosis patients. In contrast to a previous finding [12] we could not verify that PPARγ expression is reduced in whole BAL cells or AM from patients with sarcoidosis. An explanation for this difference could be clinical differences in the study populations, since PPARγ gene expression in BAL cells seems only to be reduced in patients with severe and treatment requiring sarcoidosis [13]. This may affect the T-cell driven inflammation, since deletion of PPARγ in alveolar macrophages is associated with Th1 pulmonary inflammatory response [45].

Our results suggest that the reduced expression of PPARα and PPARγ contribute to the persistent T-cell driven immunoresponse noted in non-resolving sarcoidosis, common in non-LS patients. The lower expression of these transcription factors in T cells from non-LS patients is in agreement with our previous demonstration of higher IFNγ expression in BAL T cells in that patient group. It is worth mentioning that studies have not evidently demonstrated that corticosteroids or any other therapy prevents progression or fibrosis [46,47]. The role of TNF-blocking agents in treating sarcoidosis is less clear and modulation of one cytokine is unlikely to resolve all aspects of the disease [15]. PPARs could be possibly novel targets for regulating the inflammatory processes in sarcoidosis, and continued research may result in an alternative approach to the treatment. Indeed, a clinical trial showed that short-term treatment with rosiglitazone (PPARγ agonist) is effective in patients with mild to moderately active ulcerative colitis [48]. In addition, an investigative clinical trial to explore the effect of rosiglitazone, has shown modest improvements in lung function measurements in a group of smokers with mild to moderate asthma [49]. Since fibrate drugs such as gemfibrozil are ligands for PPARα, the reduced expression of PPARα in T cells of sarcoidosis patients without LS may suggest that this class of drugs may also become candidate for treatment of sarcoidosis.

## Additional files

Additional file 1: Figure S1. Flow cytometric analysis of PPARs expression in BAL CD3^+^ CD8^+^ T cells. (a) PPARα. (b) PPARβ/δ. (c) PPARγ. HC (n = 6); LS patients (n =4); non-LS patients (n = 4). The boxes show median (25th-75th percentiles) values and the whiskers show minimum and maximum values. Each symbol represents an individual patient. Additional file 2: Figure S2. Flow cytometric analysis of PPARs expression in peripheral blood CD3^+^ CD8^+^ T cells. (a) PPARα. (b) PPARβ/δ. (c) PPARγ. HC (n = 6); LS patients (n = 4); non-LS patients (n = 4). The boxes show median (25th-75th percentiles) values and the whiskers show minimum and maximum values. Each symbol represents an individual patient. Additional file 3: Figure S3. Comparison of PPARα, PPARβ/δ and PPARγ expression between BAL and blood CD4^+^ T cells in the same individual. MFI: median fluorescence intensity. (*p <0.05, Wilcoxon’s signed ranks test). HC (n = 6); LS patients (n = 4); non-LS patients (n = 4).
